# Combined wIRA-Hyperthermia and Hypofractionated Re-Irradiation in the Treatment of Locally Recurrent Breast Cancer: Evaluation of Therapeutic Outcome Based on a Novel Size Classification

**DOI:** 10.3390/cancers12030606

**Published:** 2020-03-06

**Authors:** Markus Notter, Andreas R. Thomsen, Mirko Nitsche, Robert M. Hermann, Hendrik A. Wolff, Gregor Habl, Karin Münch, Anca-L. Grosu, Peter Vaupel

**Affiliations:** 1 Department of Radiation Oncology, Lindenhofspital Bern, 3012 Bern, Switzerland; markus.notter@lindenhofgruppe.ch (M.N.); karin.muench@lindenhofgruppe.ch (K.M.); 2 Department of Radiation Oncology, Medical Center, University of Freiburg, 79106 Freiburg, Germany; andreas.thomsen@uniklinik-freiburg.de (A.R.T.); anca.grosu@uniklinik-freiburg.de (A.-L.G.); 3 German Cancer Consortium (DKTK), Partner Site Freiburg and German Cancer Research Center (DKFZ), 69120 Heidelberg, Germany; 4 Center for Radiotherapy and Radiooncology Bremen and Westerstede, 28239 Bremen, Germany; nitsche@strahlentherapie-bremen.com (M.N.); hermann@strahlentherapie-westerstede.com (R.M.H.); 5 Department of Radiology, Nuclear Medicine and Radiotherapy, Radiology Munich, 80333 Munich, Germany; h.wolff@strahlentherapie-muenchen.eu (H.A.W.); g.habl@strahlentherapie-muenchen.eu (G.H.); 6 Department of Radiation Oncology, Medical Center, University of Regensburg, 93053 Regensburg, Germany; 7 Klinikum rechts der Isar, Technical University of Munich, 81675 Munich, Germany

**Keywords:** locally recurrent breast cancer, LRBC, novel size classification, wIRA hyperthermia, superficial hyperthermia, re-irradiation, clinical outcome, toxicity

## Abstract

Effective tumor control in patients suffering from unresectable locally recurrent breast cancer (LRBC) in pre-irradiated areas can be achieved by re-irradiation combined with superficial hyperthermia. Using this combined modality, total re-irradiation dose and toxicity can be significantly reduced compared to conventionally fractionated treatment schedules with total doses of 60–66 Gy. Applying contact-free, thermography-controlled water-filtered infrared-A superficial hyperthermia, immediately followed by hypofractionated re-irradiation, consisting of 4 Gy once per week up to a total dose of 20 Gy, resulted in high overall response rates even in large-sized tumors. Comparability of clinical data between different combined Hyperthermia (HT)/Radiotherapy (RT) treatment schedules is impeded by the highly individual characteristics of this disease. Tumor size, ranging from microscopic disease and small lesions to large-sized cancer en cuirasse, is described as one of the most important prognostic factors. However, in clinical studies and analyses of LRBC, tumor size has so far been reported in a very heterogeneous way. Therefore, we suggest a novel, simple and feasible size classification (rClasses 0–IV). Applying this classification for the evaluation of 201 patients with pre-irradiated LRBC allowed for a stratification into distinct prognostic groups.

## 1. Introduction

There is increasing interest in the use of superficial hyperthermia (sHT) in the treatment of locally recurrent breast cancer (LRBC). In the case of nonresectable lesions, previous irradiation and resistance to systemic therapy, combined hyperthermia (HT) and re-irradiation (re-RT) with reduced total doses is a promising option for tumor control if re-irradiation with therapeutically effective doses alone is limited by cumulative toxicity [1,2]. Moreover, the option of systemic therapy (e.g., endocrine, cytostatic or targeted treatment) may often be excluded due to resistance or expected toxicity, as discussed by Oldenborg et al. [3]. Hyperthermia has proven to be an effective radiosensitizer [4,5]. This is of high relevance in the treatment of locally recurrent breast cancers with significantly poorer vascularization and oxygenation status than in the respective primaries [6,7]. To achieve optimal synergy, the time interval between HT and re-RT should be as short as possible [1,8,9,10], and tissue temperature should be high enough to block DNA-repair [11]. In addition, radiotherapy (RT)-induced antitumor immune responses may be enhanced by combination with HT [12,13], as well as by hypofractionated treatment schedules [14]. A meta-analysis and systematic review based on randomized clinical trials, single-arm studies and retrospective analyses have proven the efficacy of sHT combined with RT in the treatment of LRBC [15]. This has been confirmed by several recent reviews [16,17,18,19,20]. Hyperthermia can also be combined with adjuvant re-RT after surgery of local recurrences in the case of “microscopic disease” upon R1 resection or high-risk situations due to close surgical margins [21,22,23].

The size of tumor lesions has often been described as the most significant disease-related factor for tumor response and local control rates (e.g., [24]). LRBC in pre-irradiated areas ranges from microscopic disease and small lesions in patients to be treated with curative intent up to cancer en cuirasse where palliative treatment might deliberately be restricted to areas directly affecting quality of life (QoL), e.g., ulceration, bleeding, infection, pain, obstruction or constriction. Nevertheless, reporting of the size of tumor lesions is often unsatisfactory or occurs using completely different classifications, thus impeding the comparability of published data. Classical TNM classification is not appropriate for LRBC.

A stratification should be as simple as possible based on precise and reproducible definitions relevant for therapeutic decision-making (e.g., palliative vs. potentially curative approach; total dose and fractionation). After treatment of 201 patients with microscopic and macroscopic pre-irradiated LRBC, the majority presenting with large-sized lesions, we suggest a novel size classification of LRBC and describe the treatment results based on this classification.

## 2. Patients and Methods

**Figure 1:**
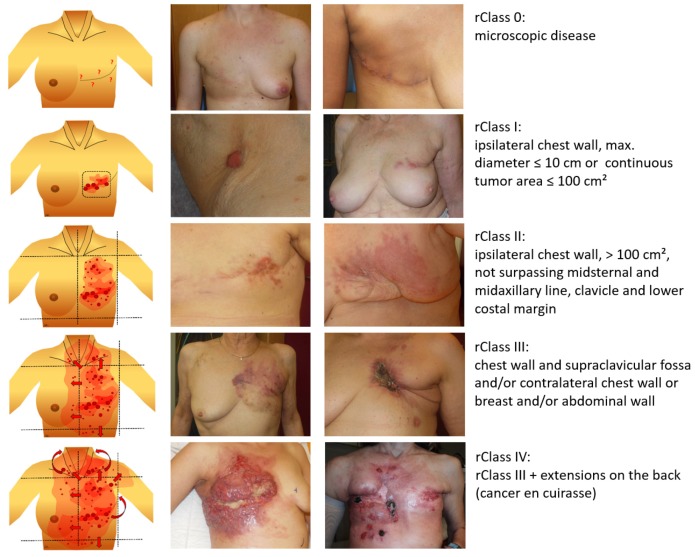
Suggested novel size classification for locally recurrent breast cancer (rClasses 0–IV).

In this evaluation, we suggest and apply novel size classes of LRBC (rClasses), as shown in Figure 1. 201 LRBC patients (199 females, 2 males) with 284 regions treated with thermography-controlled sHT using water-filtered infrared-A (wIRA) immediately followed by re-RT from 09/2009–09/2019 in standard routine use are retrospectively analyzed (66 patients from Hôpital Cantonal, La Chaux-de-Fonds, Switzerland; 106 patients from Lindenhofspital, Bern, Switzerland; 17 patients from the Medical Center, University of Freiburg, Germany; 7 patients from Radiology Munich, Munich, Germany; 5 patients from the Center for Radiotherapy and Radiooncology Bremen and Westerstede, Bremen, Germany). Ethic votum was not required for retrospective analyses since patients have been treated in standard routine use. 

Table 1 summarizes baseline patient and tumor characteristics at the time of initial diagnosis of breast cancer. Table 2 shows baseline tumor characteristics at first presentation for treatment using wIRA-HT combined with re-RT; 135/170 (79%) of the patients with macroscopic disease presented with large-sized tumors (rClass II–IV). Tumor size of 142 patients was measured in cm^2^ (12–4200; median 555), and of 39 patients in cm^3^ (3–2100; median 36). 

**Figure 2:**
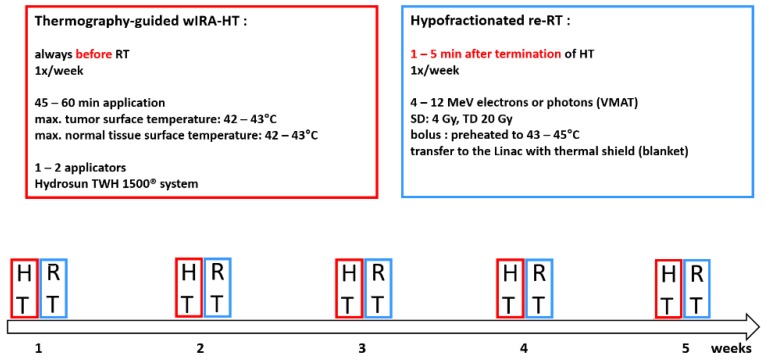
Treatment protocol: wIRA-HT immediately (i.e., within 1–5 min) followed by radiotherapy, 5 × 4 Gy, 1×/week.

The treatment protocol (Figure 2) consisted of weekly contact-free, thermography-controlled wIRA-sHT (for 45–60 min), immediately followed by hypofractionated re-RT (4 Gy once per week up to a total dose of 20 Gy), as described in detail earlier [1,25]. Seventeen patients (8%) received a total dose <20 Gy; 7 patients (3%) received a total dose >20 Gy, of them 1 with a modified scheme of 5 × 5 Gy (1 ×/week) split up in 2 × 2.5 Gy. Since 07/2017 all patients were treated with the medical device hydrosun^®^-TWH1500 (Hydrosun Medizintechnik, Müllheim, Germany). The computer-based closed feedback system of this device was set to a maximum skin surface temperature between 42.5 and 43 °C, resulting in tissue temperatures >40 °C in a depth of approximately 15 mm, and >39 °C in a depth of approximately 30 mm [25].

Local tumor response (complete response (CR), partial response (PR), no change (NC), progressive disease (PD)) was assessed clinically and—whenever possible—by imaging or biopsy, at the completion of treatment and at follow ups every 6 to 12 weeks in the first year, and every 6 to 12 months thereafter, according to WHO criteria (CR = complete clinical disappearance of all detectable disease in the treatment fields observed, PR = decrease > 50%, NC = decrease < 50% or increase < 25%, PD = increase > 25%). Local control (LC) after CR and local progression-free survival after PR were defined as absence of new local tumor progression in or at the border of the treatment field.

Acute and chronic toxicity was registered according to RTOG/EORTC criteria and to corresponding burning grades for hyperthermia-related side effects.

Tumor response, LC after CR, local progression-free survival after PR and overall survival (OS) were evaluated and correlated with tumor size according to the suggested classification. In addition, the impact of tumor growth pattern (lymphangiosis carcinomatosa, ulceration, tumor nodules), triple negative disease, time interval between first breast cancer treatment and first local relapse, menopausal status and presence of metastasis were assessed.

## 3. Results

**Figure 3:**
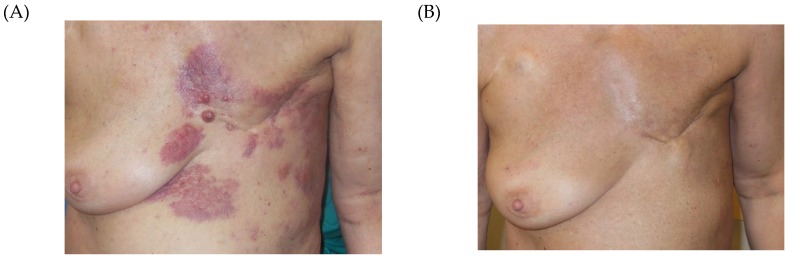
Treatment response of a patient with LRBC, rClass III. (**A**) April 17, 2018: extended locally recurrent breast cancer patient before retreatment (rClass III, 2 treatment fields), (**B**) November 12, 2018: after 2 series of HT/RT, 5 × 4 Gy, 1×/week.

**Figure 4:**
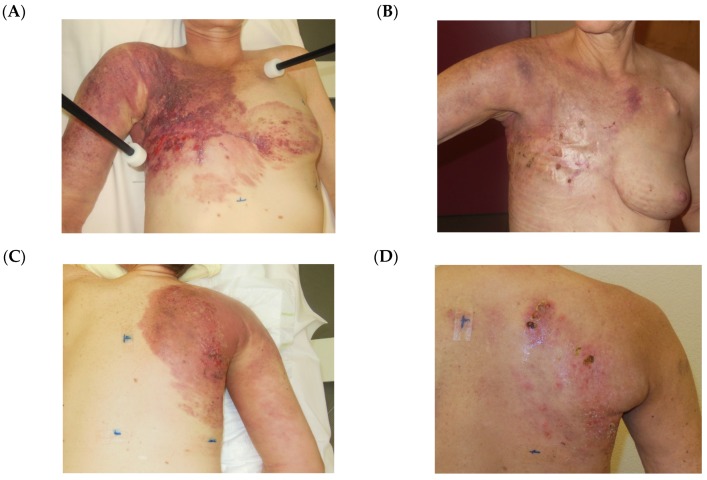
Treatment response of a patient with LRBC, rClass IV. (**A**) July 10, 2018: patient with very extended locally recurrent breast cancer “cancer en cuirasse”, rClass IV, before combined treatment of the anterior chest wall. (**B**) November 12, 2018: 2 months after HT/RT, 5 × 4 Gy 1×/week. (**C**) August 20, 2018: same patient: tumor situation on her back/right shoulder just before combined treatment. (**D**) November 12, 2018: 3 weeks after after HT/RT, 5 × 4 Gy 1×/week.

Tumor response rates are shown in Table 3. CR rate decreased with increasing tumor size: 76% in rClass I, 61% in rClass II and 36% in rClass III. In rClass IV only 1 CR was achieved. Correspondingly, PR rate increased with tumor size. Overall clinical response OR (CR + PR) was 100% in rClass I, 97% in rClass II, 97% in rClass III and 85% in rClass IV. Treatment responses of two patients were exemplarily shown in Figure 3 (CR of a patient with LRBC, rClass III) and Figure 4 (PR of a patient with LRBC, rClass IV).

Local control (LC) after CR during lifetime is shown in Table 4. LC rate of rClass 0 patients (microscopic disease) was considerably affected by “lost to follow-up” (LFU). rClass I patients showed best LC rate of macroscopic disease. There was no obvious difference between LC rate of rClass II and III. Patients presenting with lymphangiosis carcinomatosa, and ulcerations had lower CR and LC rates.

Local progression-free survival after PR during lifetime is shown in Table 5. There is no major difference between rClasses I, II, and III. In rClass IV, 71% of the patients remained locally stable without tumor progression during lifetime. 

Re-recurrences after CR and new local progression after PR are presented in Table 6. The majority of these patients could successfully be retreated with the same treatment schedule. Recurrences in chest wall areas outside the initial pre-irradiated treatment field (20 fields after CR and 34 fields after PR) were considered as distant failures and could equally be treated using HT/RT.

**Figure 5:**
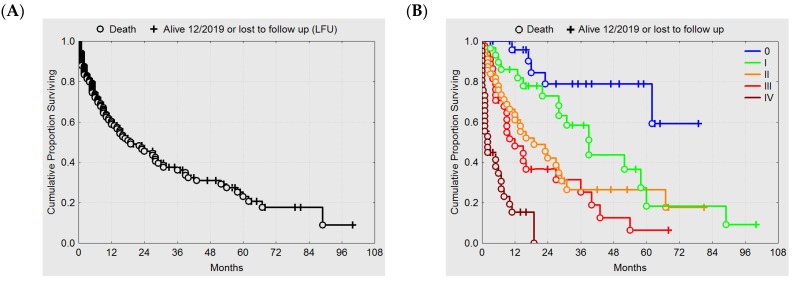

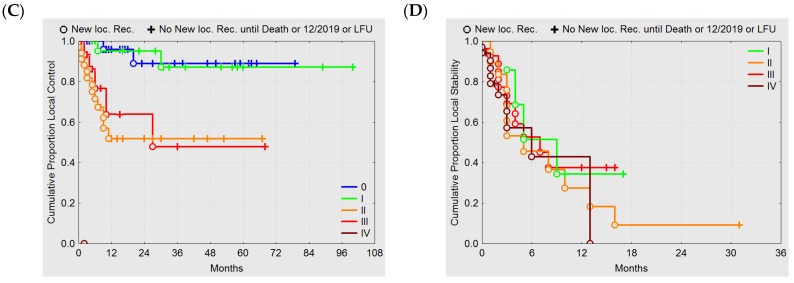
Kaplan-Meier estimates of (**A**) overall survival of all patients (*n* = 201), (**B**) overall survival stratified by rClasses 0-IV, (**C**) local control after CR stratified by rClasses 0-IV, and (**D**) progression-free interval after PR stratified by rClasses I-IV.

Using Kaplan–Meier estimates, overall survival (OS) of the total population (*n* = 201) is presented in Figure 5A. Stratification by novel rClasses 0–IV is shown in Figure 5B and reveals distinct differences in OS between rClasses, with the exception of rClasses II and III. LC after CR is shown in Figure 5C. LC of the only patient belonging to rClass IV is depicted in the left lower corner. Data for local progression-free survival after PR are similar in all rClasses (Figure 5D), indicating that even patients with very extended lesions may benefit from HT/re-RT. 

**Figure 6:**
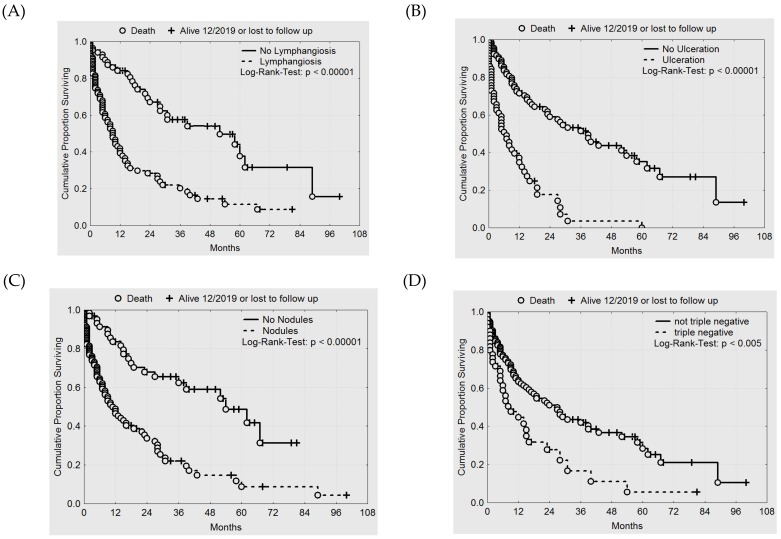
Kaplan-Meier estimates of (**A**) survival and lymphangiosis carcinomatosa, (**B**) survival and ulceration, (**C)** survival and formation of nodules within diffuse lesions, and (**D**) survival and triple negative disease.

Lymphangiosis carcinomatosa (Figure 6A), ulceration (Figure 6B), formation of nodules mainly arising in diffuse lesions (Figure 6C) and triple negative disease (Figure 6D) were strong prognostic factors in our analysis.

**Figure 7:**
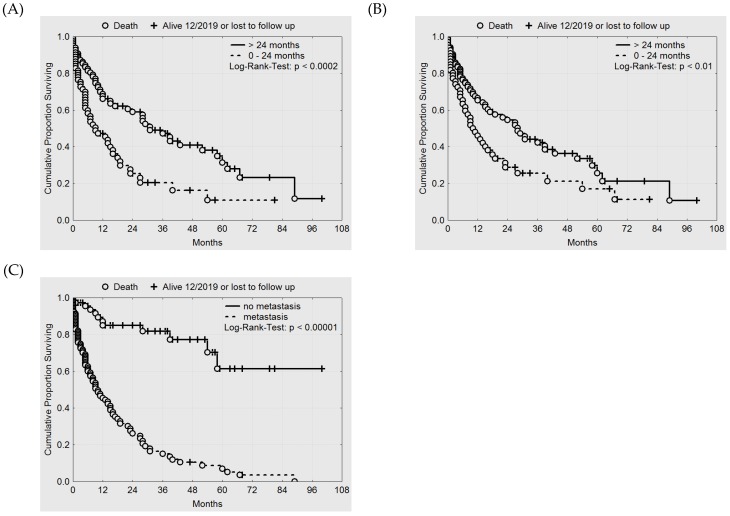
Kaplan-Meier estimates of (**A**) survival and time interval between initial treatment and recurrence, (**B**) survival and time interval between initial RT and re-RT, and (**C**) survival and metastatic status at onset of HT/re-RT or thereafter.

In addition, short time intervals between initial treatment and recurrence (Figure 7A), short time intervals between initial RT and re-RT (Figure 7B) and presence of distant metastasis at the onset of HT/re-RT or thereafter (Figure 7C) are significant negative prognostic factors. Thus, patients without metastasis have a favorable prognosis, justifying a treatment with curative intent.

There was no difference in survival between pre- and postmenopausal patients.

Treatment toxicity using thermography-controlled wIRA-HT/re-RT was limited to grade 1 and 2 toxicities, as seen in Table 7. As to hyperthermia-specific side-effects, no burns and blisters have been observed so far, with the exception of one blister above an implanted nipple of a reconstructed breast.

## 4. Discussion

Tumor size of LRBC has frequently been described as a significant prognostic factor for clinical outcomes (e.g., [26]). Additionally, tumor size has an important impact on the risk/benefit assessment of re-RT alone with conventional doses. However, so far this has been assessed and reported in a largely heterogenous way. 

In a retrospective analysis of 414 patients, Oldenborg et al. [24] separated four size groups, namely <3 cm, 3–5 cm, 5–10 cm and >10 cm. In their analysis, 48% of the patients were assigned to the group >10 cm. In a multivariate analysis of prognostic factors for local control (LC), tumor size was significant. Lee et al. [26] classified lesions into nodular tumors subdivided in ≤3 cm and >3 cm max. Diameter versus diffuse tumors defined as areas >20 cm^2^, the latter showing the poorest local control. In a retrospective analysis of 36 patients, Dharmaiah et al. [27] described tumor size by volume (median 573.9 cc, ranging from 11.7 to 3619.8 cc), emphasizing the inclusion of large volumes of disease. The response rates were CR 47%, PR 14%, SD 31% and PD 8%, respectively. However, in the case of large, spotted and irregularly shaped lesions, the measurement in diameter (cm), or area (cm^2^), or volume (cm^3^ or cc) might be difficult and sometimes almost impossible. Oldenborg et al. [3] published a subgroup analysis of 169 out of 414 patients presenting with “cancer en cuirasse”, defined as a lesion ≥ ½ of the ipsilateral chest wall. Response rates were as follows: CR 30%, PR 42%, SD 22% and PD 6% (compared to CR 58%, PR 28%, SD 12% and PD 2% of the total group reported [20]). The subgroup was further separated in two groups of ≥½ to <¾ and ≥¾ chest wall, which again differed significantly in the overall response rates.

Considering the relevant literature on HT/re-RT in the treatment of LRBC, the presentation of response rates using accumulated data of all patients without stratification by tumor size and other prognostic factors, is, up to now, not satisfactory. In the systematic review and meta-analysis of Datta et al. [15], only CR rate was chosen as the decisive outcome parameter; 779 pre-irradiated patients from 16 single-or two-arm studies were treated with re-RT/HT, achieving a CR rate of 66.6%. Oldenborg et al. noted that “these relatively high response rates resulted from the inclusion of studies on small, single lesions” [3]. In the evaluation of the first 73 patients treated from September 2009 to July 2015 with wIRA-HT/re-RT, Notter et al. reported a 61% CR remission rate of macroscopic disease [1]. In the evaluation presented in this paper, the CR rate of 170 patients with macroscopic disease treated from September 2009 to September 2019 was 43%. In this time period, the percentage of patients with large-sized tumors as well as with lymphangiosis carcinomatosa and ulcerations continuously increased, whereas the percentage of small-sized tumors decreased. This is confirmed by interim analyses of 102 patients [28], and 140 patients [29]. 

Taking into account these obvious problems, we suggest and apply in this analysis a clear, conclusive and applicable classification of locally recurrent breast cancer, which may improve comparability of data. Besides tumor size, the presence of lymphangiosis carcinomatosa and ulcerations should always be indicated. In contrast, we deliberately renounced the differentiation between “nodular” and “diffuse”. rClass I tumors can consist of single nodules or a small area of diffuse lesion, whereas in a generally diffuse situation of rClass II–IV tumors nodules may additionally be present even as a negative prognostic factor (see Figure 6C). 

This novel classification allows one to stratify patients into distinct prognostic groups, ranging from a high probability to achieve CR (rClass I) to the most advanced group (rClass IV), where PR can be estimated as the best possible treatment outcome with tremendous impact on QoL. Therefore, the classification may also be helpful for the general decision-making between curative and palliative intent, along with other significant influencing factors (e.g., lymphangiosis carcinomatosa, ulceration, hormone receptor status, occurrence of nodular masses, distant metastasis, time interval between primary treatment and recurrence). 

Expectedly, rClass 0 and rClass I show the best local control after CR, and rClass IV the worst overall survival (OS). Interestingly, in spite of large difference in response rate, Classes II and III show no major difference in local control after CR and in OS. 

Kaplan–Meier estimates comparing patients with and without distant metastasis may provide evidence for the benefit in survival that can be achieved by HT/re-RT in patients without metastasis (Figure 7C). In six patients treated for macroscopic disease, there is—to date—no evidence of disease (NED) for more than one year (median 49 months, range 13–100 months). Interestingly, these data include two patients of rClass I, three patients of rClass II, and one patient of rClass III. In the case of PR, local stabilization during the lifetime could be achieved in more than half of the patients and could be kept for more than one year in some patients (see Figure 5D). 

In LRBC patients, new randomized clinical trials (RCT) comparing HT/re-RT versus best supportive care (without tumor-directed therapy) are presumably not feasible. Besides ethical considerations, patients with heavily pretreated macroscopic disease may refuse randomization and insist on the immediate start of treatment with the intention of effective tumor control. RCTs comparing combined HT/re-RT with reduced doses versus re-RT alone using conventional doses would either exclude or compromise those patients who are at risk of unacceptable cumulative toxicity. Moreover, the highly individual differences in lesion sizes, type and number of pre-treatments, resistances to other therapies, comorbidities, etc., impede randomization into comparable groups.

Given the current level of evidence, the extreme suffering of the patients and the lack of other therapeutic options with comparable risk/benefit assessment and evidence, superficial HT/re-RT can be considered as treatment of choice for inoperable, pre-irradiated LRBC. Accordingly, in the leading German breast cancer guideline [30], combined HT/RT has the highest level of recommendation of all listed therapeutic options for non-curative cases of LRBC, as well as for adjuvant therapy after mastectomy following local recurrence after breast conserving therapy.

Nevertheless, there is a need for prospective single-arm studies, as well as retrospective analyses of routine use with relatively high numbers of patients to find optimal schemes of RT doses, fractionation and target temperatures of combined HT/re-RT treatment, to overcome heterogeneity in applied schedules and the lack of generally accepted treatment standards as criticized earlier [31]. A generally accepted size classification is expected to be helpful for the assessment of outcome data within a study and the data comparability of different studies. Our suggestion aims to initiate a discussion about the best classification and stratification of LRBC patients.

The hypofractionated treatment schedule and heating technique described in this analysis leads to satisfying responses and local control rates. Treatment-related toxicity, especially acute and chronic skin damage, could be significantly reduced compared to other schedules and techniques described before [3,21,24,26,27]. 

## 5. Conclusions

The novel classification presented here is well feasible in the analysis of patients with pre-irradiated LRBC and allows for the assessment of chances and limitations of combined HT/re-RT in the treatment of different tumor sizes. It may improve comparability of data and stratification of prognostic groups, thus helping to guide the decision between curative and palliative intent. Using this classification, the retrospective analysis of 201 patients treated with contact-free, thermography-controlled wIRA-HT immediately followed by hypofractionated re-RT (5 × 4 Gy, 1×/week) results in a high clinical overall response rate and a satisfying local control rate even in large-sized tumors of LRBC. Low toxicity allows for repeated re-irradiations in case of re-recurrences.

## Figures and Tables

**Table 1:** Baseline patient and tumor characteristics at initial diagnosis of breast cancer. n.a. = not applicable.

Characteristics	Subgroups	No. of Patients
Median age (years)	54 (range: 28–91)	201
Menopausal status	premenopausal	57
	perimenopausal	29
	postmenopausal	113
	n.a.	2
T-stage	T1	58
	T2	78
	T3	30
	T4	34
	TX	1
Lymph node involvement	N0	65
	N1	71
	N2	35
	N3	28
	NX	2
Distant metastasis	M1	185
	M1	16
Histological grading	G1	16
	G2	80
	G3	105
Resection status	R0	186
	R1	15
Estrogen receptor expression	positive	124
	negative	27
Progesterone receptor expression	positive	109
	negative	42
Her2/new expression	amplified	47
	missing	74
	unknown	30
Triple negative		50

**Table 2:** Baseline patient and tumor characteristics at onset of hyperthermia/re-irradiation.

Characteristics	Median (Range)	No. of Patients
Total number of patients		201
No. of tumor regions treated		284
Age (years)	65 (31–102)	
Interval between initial RT and re-RT (months)	47 (0–389)	
Presence of distant metastasis		91
Number of previous recurrences	2 (1–11)	
Previous RT-dose (Gy)	60 (20–139)	
Re-irradiation dose (Gy)		
4		1
8		2
12		6
16		8
20		177
24		5
25		1
28		1
No. of previous chemotherapies		
0		32
1		20
2		33
>2		116
No. of previous hormone therapies		
0		83
1		34
2		27
>2		57
No. of previous resections		
0		10
1		88
2		59
>2		44
Tissue transfer		
None		157
Meshgraft/skin transplants/reconstruction, etc.		44
Anatomic site at the time of recurrence		
Breast		18
Chest wall		131
Regional lymph nodes		22
Regional lymph nodes and chest wall		30
LRBC extension (classification)		
Microscopic disease:		
rClass 0		31
Macroscopic disease:		170
rClass I		29
rClass II		56
rClass III		44
rClass IV		41
Gross tumor volume (cm^3^)	36 (3–2100)	39
Tumor size (cm^2^)	555 (12–4200)	142
Lymphangiosis carcinomatosa		
no		86
yes		115
Ulceration		
no		131
yes		70

**Table 3:** Tumor response.

Patients	rClass 0	Macroscopic All	Macroscopic/Size				Macroscopic/Other Factors	
			rClass I	rClass II	rClass III	rClass IV	Lymphang	Ulceration
	31 (100%)	170 (100%)	29 (100%)	56 (100%)	44 (100%)	41 (100%)	115 (100%)	70 (100%)
**CR**		73 (43%)	22 (76%)	34 (61%)	16 (36%)	1 (2%)	41 (36%)	12 (17%)
**PR**		88 (52%)	7 (24%)	20 (36%)	27 (61%)	34 (83%)	68 (59%)	52 (74%)
**NC**		6 (4%)		2 (3%)		4 (10%)	4 (3%)	4 (6%)
**PD**		3 (2%)			1 (2%)	2 (5%)	2 (2%)	2 (3%)

**Table 4:** Local control (LC) during lifetime and infield/border re-recurrences after complete response (CR). LFU = Lost to follow up.

Patients	rClass 0	Macroscopic All	Macroscopic/Size Classes				Macroscopic/Other Factors	
			rClass I	rClass II	rClass III	rClass IV	Lymphang	Ulceration
	31 (100%)	73 (100%)	22 (100%)	34 (100%)	16 (100%)	1 (100%)	41 (100%)	12 (100%)
**LC**	21 (68%)	49 (67%)	17 (77%)	21 (62%)	11 (69%)		24 (59%)	7 (58%)
**Local re-rec**	2 (6%)	21 (29%)	2 (9%)	13 (38%)	5 (31%)	1 (100%)	17 (41%)	5 (42%)
**LFU**	8 (26%)	3 (4%)	3 (14%)					

**Table 5:** Progression free rate during lifetime and new infield/border progression after partial response (PR).

Patients	Macroscopic All	Macroscopic/Size				Macroscopic/Other Factors	
		rClass I	rClass II	rClass III	rClass IV	Lymphang	Ulceration
	88 (100%)	7 (100%)	20 (100%)	27 (100%)	34 (100%)	68 (100%)	52 (100%)
**Locally progression-free** **New local progression**	48 (55%) 40 (45%)	3 (43%) 4 (57%)	8 (40%) 12 (60%)	14 (52%) 13 (48%)	23 (71%) 11 (29%)	37 (56%) 31 (44%)	27 (54%) 25 (46%)

**Table 6:** Macroscopic disease—Analysis of re-recurrences after complete response (CR) and new progression after partial response (PR).

Characteristics	Re-Recurrences after CR	New Local Progression after PR
Number of patients	21	40
Number of re-recurrences (fields)	25	53
infield border	7 18	27 26
Number of patients treated with re-HT/re-re-RT	19	24
CR after first re-HT/re-re-RT	13	2
PR after first re-HT/re-re-RT	6	16
NC after first re-HT/re-re-RT	0	4
PD after first re-HT/re-re-RT	0	2

**Table 7:** Toxicity of water-filtered infrared-A hyperthermia (wIRA-HT) and re-irradiation (re-RT).

No. of Patients	201 (100%)
No acute side effects Acute side effects Radiodermatitis Grade I Radiodermatitis Grade II Scurfs Hyperpigmentation Burn with blistering	114 (57%) 87 (43%) 65 4 10 64 1
No chronic side effects Chronic side effects Grade 1: hyperpigmentation Grade 2: new teleangiectasia	145 (72%) 56 (28%) 53 7

## References

[1] Notter M., Piazena H., Vaupel P. (2017). Hypofractionated re-irradiation of large-sized recurrent breast cancer with thermography-controlled, contact-free water-filtered infra-red-A hyperthermia: A retrospective study of 73 patients. Int. J. Hyperth..

[2] Merino T., Tran W.T., Czarnota G.J. (2015). Re-irradiation for locally recurrent refractory breast cancer. Oncotarget.

[3] Oldenborg S., Rasch C.R.N., Os R.V., Kusumanto Y.H., Oei B.S., Venselaar J.L., Heymans M.W., Zum Vörde Sive Vörding P.J., Crezee H., Tienhoven G.V. (2017). Reirradiation + hyperthermia for recurrent breast cancer en cuirasse. Strahlenther. Onkol..

[4] Horsman M.R., Overgaard J. (2007). Hyperthermia: A potent enhancer of radiotherapy. Clin. Oncol. (R. Coll. Radiol.).

[5] Elming P.B., Sørensen B.S., Oei A.L., Franken N.A.P., Crezee J., Overgaard J., Horsman M.R. (2019). Hyperthermia: The optimal treatment to overcome radiation resistant hypoxia. Cancers.

[6] Vaupel P., Höckel M., Mayer A. (2007). Detection and characterization of tumor hypoxia using pO_2_ histography. Antioxid. Redox Signal..

[7] Vaupel P., Briest S., Höckel M. (2002). Hypoxia in breast cancer: Pathogenesis, characterization and biological/therapeutic implications. Wien. Med. Wschr..

[8] Thomsen A.R., Aldrian C., Niedermann G., Grosu A.L., Vaupel P., Lund P.G. Differential effects of 42 °C-hyperthermia on radiation response of breast cancer spheroids vs. normal human skin explants. Proceedings of the 36th Annual Meeting of the Society for Thermal Medicine (STM).

[9] Overgaard J. (1980). Simultaneous and sequential hyperthermia and radiation treatment of an experimental tumor and its surrounding normal tissue in vivo. Int. J. Radiat. Oncol. Biol. Phys..

[10] van Leeuwen C.M., Oei A.L., Chin K.W.T.K., Crezee J., Bel A., Westermann A.M., Buist M.R., Franken N.A.P., Stalpers L.J.A., Kok H.P. (2017). A short time interval between radiotherapy and hyperthermia reduces in-field recurrence and mortality in women with advanced cervical cancer. Radiat. Oncol..

[11] Oei A.L., Kok H.P., Oei S.B., Horsman M.R., Stalpers L.J.A., Franken N.A.P., Crezee J. (2020). Molecular and biological rationale of hyperthermia as radio-and chemosensitizer. Adv. Drug Deliv. Rev..

[12] Frey B., Weiss E.M., Rubner Y., Wunderlich R., Ott O.J., Sauer R., Fietkau R., Gaipl U.S. (2012). Old and new facts about hyperthermia-induced modulations of the immune system. Int. J. Hyperth..

[13] Dewhirst M.W., Vujaskovic Z., Jones E., Thrall D. (2005). Re-setting the biologic rationale for thermal therapy. Int. J. Hyperth..

[14] Kötter B., Frey B., Winderl M., Rubner Y., Scheithauer H., Sieber R., Fietkau R., Gaipl U.S. (2015). The in vitro immunogenic potential of caspase-3 proficient breast cancer cells with basal low immunogenicity is increased by hypofractionated irradiation. Radiat. Oncol..

[15] Datta N.R., Puric E., Klingbiel D., Gomez S., Bodis S. (2016). Hyperthermia and radiation therapy in locoregional recurrent breast cancers: A systematic review and meta-analysis. Int. J. Radiat. Oncol. Biol. Phys..

[16] Marta G.N., Hijal T., Carvalho H.D.A. (2017). Reirradiation for locally recurrent breast cancer. Breast.

[17] Wadasadawala T., Vadgaonkar R., Bajpai J. (2017). Management of isolated locoregional recurrences in breast cancer: A review of local and systemic modalities. Clin. Breast Cancer.

[18] Kaidar-Person O., Oldenborg S., Poortmans P. (2018). Re-irradiation and hyperthermia in breast cancer. Clin. Oncol..

[19] Arslan S.A., Ozdemir N., Sendur M.A., Eren T., Ozturk H.F., Aral I.P., Delikgoz E.D., Inan G.A. (2018). Hyperthermia and radiotherapy combination for locoregional recurrences of breast cancer: A review. Breast Cancer Manag..

[20] Youssef I. (2019). Hyperthermia for Chest Wall Recurrences.

[21] De-Colle C., Weidner N., Heinrich V., Brucker S., Hahn M., Macmillan K., Lamprecht U., Gaupp S., Voigt O., Zips D. (2019). Hyperthermic chest wall re-irradiation in recurrent breast cancer: A prospective observational study. Strahlenther. Onkol..

[22] Oldenborg S., Os R.V., Oei B., Poortmans P. (2019). Impact of technique and schedule of reirradiation plus hyperthermia on outcome after surgery for patients with recurrent breast cancer. Cancers.

[23] Linthorst M., Geel A.N.V., Baaijens M., Ameziane A., Ghidey W., Rhoon G.C.V., van der Zee J. (2013). Re-irradiation and hyperthermia after surgery for recurrent breast cancer. Radiother. Oncol..

[24] Oldenborg S., Griesdoorn V., Os R.V., Kusumanto Y.H., Oei B.S., Venselaar J.L., Paul J., Vörding Z.V.S., Heymans M.W., Kolff M.W. (2015). Reirradiation and hyperthermia for irresectable locoregional recurrent breast cancer in previously irradiated area: Size matters. Radiother. Oncol..

[25] Vaupel P., Piazena H., Müller W., Notter M. (2018). Biophysical and photobiological basics of water-filtered infrared-A hyperthermia of superficial tumors. Int. J. Hyperth..

[26] Lee H.K., Antell A.G., Perez C.A., Straube W.L., Ramachandran G., Myerson R.J., Emami B., Molmenti E.P., Buckner A., Lockett M.A. (1998). Superficial hyperthermia and irradiation for recurrent breast carcinoma of the chest wall: Prognostic factors in 196 tumors. Int. J. Radiat. Oncol. Biol. Phys..

[27] Dharmaiah S., Zeng J., Rao V.S., Zi O., Ma T., Yu K., Bhatt H., Shah C., Godley A., Xia P. (2019). Clinical and dosimetric evaluation of recurrent breast cancer patients treated with hyperthermia and radiation. Int. J. Hyperth..

[28] Notter M., Vaupel P. Re-irradiation and wIRA-hyperthermia for superficial widespread breast cancer recurrences: An update. Proceedings of the 31st Annual Meeting of the European Society for Hyperthermic Oncology (ESHO).

[29] Notter M., Vaupel P. (2018). Water-filtered infrared-A hyperthermia & re-irradiation in the treatment of recurrent breast cancer. Strahlenther. Onkol..

[30] AGO (Arbeitsgemeinschaft Gynäkologische Onkologie) Innerhalb Der Deutschen Gesellschaft Für Gynäkologie Und Geburtshilfe (DGGG) Und Der Deutschen Krebsgesellschaft e.V. (DKG) Guidelines Breast 2019, Version 2019.1, Loco-Regional Recurrence, Page 17 and 19. https://www.ago-online.de/fileadmin/ago-online/downloads/_leitlinien/2019/PDF_EN/2019E_17_Loco-Regional_Recurrence_with_References.pdf.

[31] McCormick B. (2007). Counterpoint: Hyperthermia with radiation therapy for chest wall recurrences. J. Natl. Compr. Cancer Netw..

